# Receiver operating characteristic analysis of prediction for gastric cancer development using serum pepsinogen and *Helicobacter pylori* antibody tests

**DOI:** 10.1186/s12885-017-3173-0

**Published:** 2017-03-09

**Authors:** Chisato Hamashima, Shizuka Sasazuki, Manami Inoue, Shoichiro Tsugane, for the JPHC Study Group

**Affiliations:** 1 Division of Cancer Screening Assessment and Management, Center for Social Public Health Sciences, National Cancer Center, 5-1-1 Tsukiji Chuo-ku, Tokyo, 104-0045 Japan; 2 Epidemiology and Prevention Group, Center for Public Health Sciences, National Cancer Center, 5-1-1 Tsukiji Chuo-ku, Tokyo, 104-0045 Japan; 3 Department of Global Health Policy, Graduate School of Medicine, The University of Tokyo, 7-3-1 Hongo, Bunkyo-ku, Tokyo, 113-0033 Japan

**Keywords:** Gastric cancer, *Helicobacter pylori* antibody, Serum pepsinogen, Receiver operating characteristic analysis, Cancer screening

## Abstract

**Background:**

Chronic *Helicobacter pylori* infection plays a central role in the development of gastric cancer as shown by biological and epidemiological studies. The *H. pylori* antibody and serum pepsinogen (PG) tests have been anticipated to predict gastric cancer development.

**Methods:**

We determined the predictive sensitivity and specificity of gastric cancer development using these tests. Receiver operating characteristic analysis was performed, and areas under the curve were estimated. The predictive sensitivity and specificity of gastric cancer development were compared among single tests and combined methods using serum pepsinogen and *H. pylori* antibody tests.

**Results:**

From a large-scale population-based cohort of over 100,000 subjects followed between 1990 and 2004, 497 gastric cancer subjects and 497 matched healthy controls were chosen. The predictive sensitivity and specificity were low in all single tests and combination methods. The highest predictive sensitivity and specificity were obtained for the serum PG I/II ratio. The optimal PG I/II cut-off values were 2.5 and 3.0. At a PG I/II cut-off value of 3.0, the sensitivity was 86.9% and the specificity was 39.8%. Even if three biomarkers were combined, the sensitivity was 97.2% and the specificity was 21.1% when the cut-off values were 3.0 for PG I/II, 70 ng/mL for PG I, and 10.0 U/mL for *H. pylori* antibody.

**Conclusions:**

The predictive accuracy of gastric cancer development was low with the serum pepsinogen and *H. pylori* antibody tests even if these tests were combined. To adopt these biomarkers for gastric cancer screening, a high specificity is required. When these tests are adopted for gastric cancer screening, they should be carefully interpreted with a clear understanding of their limitations.

## Background

Although the incidence of gastric cancer has decreased worldwide, it remains the fifth most common malignancy in the world [1]. Gastric cancer remains a heavy burden in Eastern Asia, South America, and a number of European countries. However, prevention and screening programs for gastric cancer particularly at the national level have not yet been established in most countries. The exceptions are Korea and Japan where gastric cancer screening programs have already been introduced [2]. Recently, the International Agency for Research on Cancer has suggested the establishment of *Helicobacter pylori* screening and eradication programs in countries with a high incidence of gastric cancer, taking the local context into consideration [3]. However, the efficacy of the screening methods used has not yet been evaluated, although they have been anticipated to reduce gastric cancer incidence. Thus, *H. pylori* screening has not yet been officially introduced either as a national and regional program.

Chronic *H. pylori* infection plays a central role in the development of gastric cancer as shown by biological and epidemiological studies [4]. In a recent study, *H. pylori* infection was reported to be associated with 90% of non-cardia gastric cancer [5]. The associations of other factors including Cag A, blood type, and lifestyle with gastric cancer have also been investigated [6–11]. Based on several risk factors related to lifestyle, prediction models for gastric cancer have been developed, and these models have demonstrated the capability of discriminating high-risk individuals [12]. The serum pepsinogen (PG) test can diagnose gastric atrophy, and it has been used for gastric cancer screening and risk stratification for gastric cancer with *H. pylori* antibody [13–17]*.* Sasazuki et al. reported that the odds ratio for gastric cancer development of *H. pylori* infection with the gastric atrophy was higher to that of *H. pylori* infection and this was lower than that of *H. pylori* infection with positive result of CagA [11]. Charvat et al. developed a prediction model for gastric cancer based on *H. pylori* infection and gastric atrophy with the risk factors related to lifestyle [18]. The strong association between gastric cancer and these risk factors suggested a high possibility of predicting gastric cancer incidence in the high-risk group detected by the serum PG and *H. pylori* antibody tests. If the future risk for gastric cancer development can be optimally clarified, appropriate preventive measures can be taken according to individual risks. These preventive measures can be made more efficient for gastric cancer screening to accurately target cancer screening subjects and decrease the screening frequency of the low-risk group. However, these results are not directly connected with primary cancer screening. To adopt these biomarkers in gastric cancer screening, both sensitivity and specificity should be assessed considering the balance of benefits and harms.

Receiver operating characteristic (ROC) analysis is a widely accepted method for selecting an optimal cut-off value for tests as well as for comparing the sensitivity and specificity of diagnostic tests [19]. Optimal sensitivity and specificity can maintain the balance of the benefits and harms of a diagnostic test. A high possibility of predicting gastric cancer incidence indicates high sensitivity, but the indication of specificity, which identifies the proportion of subjects without gastric cancer, is still unclear. A low specificity reportedly indicates a high false-positive result and this becomes harm in asymptomatic people [20]. Therefore, a high specificity is also required. However, the predictive sensitivity and specificity of these biomarkers for gastric cancer development remain unclear. In this study, we evaluated the predictive sensitivity and specificity of the *H. pylori* antibody and serum PG tests for predicting gastric cancer development by ROC analysis based on a long follow-up period.

## Methods

### Study population

The Japan Public Health Center (JPHC)-based prospective study on cancer and cardiovascular disease (JPHC study) was established in 1990. The study population was defined as all inhabitants in 27 municipalities under 9 public health centers. The study population and design of the JPHC study have been described in detail elsewhere [11]. As a whole, a population-based cohort of 61,009 men and 62,567 women was identified and followed from January 1, 1990 to December 31, 2004. Blood sample was provided voluntarily by these subjects during their health check-ups and was collected from 1990 to 1995. Although a questionnaire survey was performed at their health check-ups, there was no question related to medicines for gastric diseases which they were taking. Newly diagnosed cases of cancer were collected through local major hospitals and local cancer registries.

This study was approved by the Institutional Review Board of the National Cancer Center, Japan (Approval number: 2001-013, 14-038). Written informed consent was obtained from all the participants in the JPHC study.

### Laboratory data

The level of IgG antibodies to *H. pylori* was measured using a direct ELISA kit (E.Plate ‘Eiken’ *H. pylori* Antibody, Eiken Kagaku Co., Ltd., Tokyo, Japan). The serum levels of PG I and II were measured by two-step enzyme immunoassay using commercial kits (E.Plate ‘Eiken’ Pepsinogen I and Pepsinogen II Eiken Kagaku Co., Ltd.). All measurements were performed by a person blinded to the study. The levels of PG I, PG II, PG I/II, and *H. pylori* antibody were used for diagnosing and predicting gastric cancer development. In Japan, a combination of PG I, PG I/II, and *H. pylori* antibody measurements has been a commonly used method for stratifying the risk of gastric cancer. PG I ≤ 70 ng/mL and PG I/II ≤ 3.0 indicate chronic atrophic gastritis. *H. pylori* infection was classified as positive when the *H. pylori* antibody titer was ≥ 10 U/mL.

### Statistical analysis

ROC analysis was performed following the Hanley and McNeil’s method. The area under the curve (AUC) indicated diagnostic accuracy and defined the optimal cut-off points of the diagnostic tests. The AUC and 95% confidence interval (CI) were estimated and compared among the different biomarkers or their combination. When the highest likelihood ratio was obtained, the cut-off value for sensitivity and specificity was defined as optimal. Statistical analysis was performed using STATA 13.0 (STATA, College Station, TX, USA). All test statistics were two-tailed, and *p*-values < 0.05 were considered to indicate a statistically significant difference.

Before the main analysis for the prediction of gastric cancer development, ROC analysis for these biomarkers was performed to investigate their ability to diagnose *H. pylori* infection. *H. pylori* infection was used for determining outcome using 2 cut-off values (≥10 U/mL and ≥ 5 U/mL) and the AUCs among PG I, PG II, and PG I/II were compared. Then, gastric cancer was used for determining predictive outcome and the AUCs among PG I, PG II, PG I/II, and *H. pylori* antibody titer were compared. The AUCs were also compared among combination methods using PG I, PG II, PG I/II, and *H. pylori* antibody. Finally, the AUCs were estimated and compared between the commonly used definition of the defined value of a combination method using PG I, PG I/II, and *H. pylori* antibody. The subjects were classified into 4 groups according to the risk for gastric cancer development based on their levels of serum PG and *H. pylori* antibody at enrollment. To discriminate the positive and negative results, the following standard categories were used: PG I/II = 3.0, PG I = 70.0 ng/mL, and *H. pylori* antibody = 10.0 U/mL. Atrophic gastritis was defined on the basis of the results of a combination of PG I/II and PG I. Based on these categories, the results were divided into 4groups. The first group subjects had a “normal” PG level (negative atrophy) and were negative for *H. pylori* antibody (negative *H. pylori* infection*)*. The second group subjects had a “normal” PG level and were positive for *H. pylori* antibody. The third group subjects had an “atrophic” PG level and were positive for *H. pylori* antibody. The fourth group subjects had an “atrophic” PG level and were negative for *H. pylori* antibody.

## Results

### Target population

**Fig. 1:**
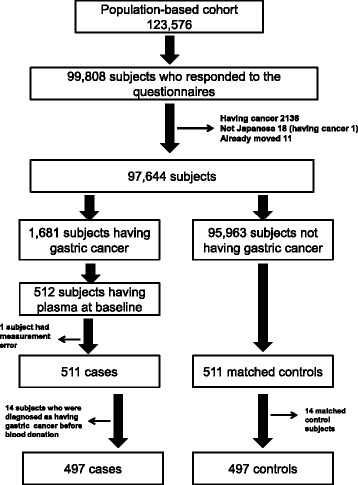
Flow chart for target group selection. In a large-scale cohort study conducted in Japan (i.e., Japan Public Health Center Study [JPHC study]), a population-based cohort of 61,009 men and 62,567 women was identified and followed from January 1, 1990 to December 31, 2004. Of 97,644 eligible subjects in the JPHC study cohort, 13,467 men and 23,278 women who donated blood samples at baseline were included. Cases of gastric cancer were extracted from the cancer registry for the JPHC study based on site. Among 1,681 cases with a histologically proven diagnosis made from 1990 to 2004, plasma at baseline was obtained from 512 cases. One case with a technical error of *H. pylori* antibody measurement and the matched control were excluded. The dataset included 511 case subjects and 511 matched control subjects. For this analysis, 14 case subjects who were diagnosed as having gastric cancer before the blood donation date and 14 matched control subjects were excluded. Thus, a total of 994 subjects (497 case subjects having gastric cancer and 497 healthy control subjects) were used for the ROC analysis in this study

We used the dataset from a nested case-control study from a large-scale cohort study conducted in Japan (i.e., JPHC study) [11]. The procedure used for the selection of the target population is shown in Fig. 1. Of 97,644 eligible subjects in the JPHC study cohort, 13,467 men and 23,278 women who donated blood samples at baseline were included. Cases of gastric cancer were extracted from the cancer registry for the JPHC study based on site. Among 1,681 cases with a histologically proven diagnosis made from 1990 to 2004, plasma at baseline had been obtained from 512 cases. The case subjects were selected individuals who were diagnosed as having gastric cancer for the follow-up periods based on information from local major hospitals and local cancer registries. For the case subjects, control subjects were selected and matched for sex, age (±3 years), blood donation date (±2 months), and fasting time at blood donation (±5 h). One case with a technical error with the *H. pylori* antibody measurement and the matched control were excluded. The dataset included 511 case subjects and 511 matched control subjects [11]. For this analysis, 14 case subjects who were diagnosed as having gastric cancer before the blood donation date and 14 matched control subjects were excluded. A total of 994 subjects (497 case subjects having gastric cancer and 497 healthy control subjects) were used for the ROC analysis in this study. The proportion of female subjects was 33.6% and the average age at participation in the cohort study was 57.5 ± 7.2 years. The average follow-up years from the blood donation date to diagnosis date of gastric cancer for case subjects was 6.1 ± 3.4 years.

### ROC analysis

When *H. pylori* infection was classified as a positive case at ≥ 10.0 U/mL, the AUC of PG I/II was 0.820 ± 0.023 (95% CI: 0.774-0.865) which was significantly higher than that of the other biomarkers (p < 0.001, Table 1). Even if the cut-off value was changed from 10.0 U/mL to 5.0 U/mL, a similar result was obtained (p < 0.001). The optimal values of PG I/II were 95.0% for sensitivity and 54.8% for specificity when the *H. pylori* antibody titer was defined as a positive case of *H. pylori* infection at ≥ 10 U/mL.

**Table 1:** Comparison of ROC areas among individual biomarkers

Outcome (Cut-off value)	ROC area (95% CI)			
	PG I/II	PG I	PG II	HP
*H. pylori* infection	0.820 ± 0.023	0.455 ± 0.022	0.186 ± 0.016	-
(HP antibody = 10.0)	(0.774–0.865)	(0.411–0.499)	(0.153–0.217)	
*H. pylori* infection	0.849 ± 0.024	0.478 ± 0.025	0.181 ± 0.018	-
(HP antibody = 5.0)	(0.801–0.896)	(0.429–0.527)	(0.146–0.216)	
Gastric cancer	0.649 ± 0.017	0.561 ± 0.011	0.434 ± 0.018	0.574 ± 0.018
	(0.615–0.683)	(0.526–0.597)	(0.400–0.469)	(0.538–0.610)

*HP Helicobacter pylori*, *PG* pepsinogen, *ROC* receiver operating characteristic, *CI* confidence interval

**Fig. 2:**
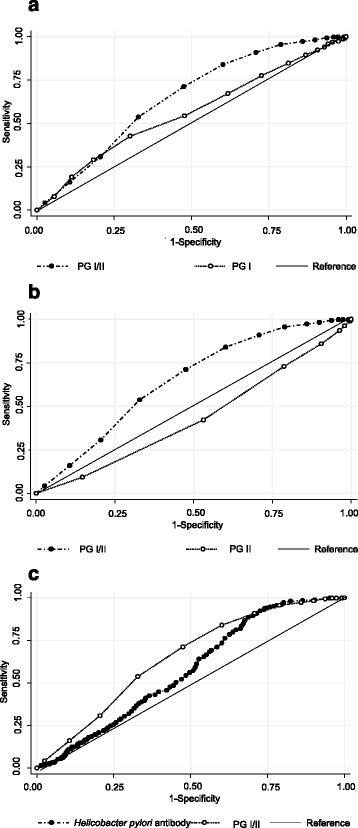
Comparison of AUCs for gastric cancer development among single tests using serum PG status and *H. pylori* antibody. The AUCs were compared among PG I, PG II, PG I/II, and *H. pylori* antibody titer. The AUCs of the following tests were compared with that of PG I/II (AUC = 0.649 ± 0.017, 95% CI: 0.615-0.683): **a**, the AUC of PG I (0.561 ± 0.011, 95% CI: 0.526-0.597) was significantly lower than that of PG I/II (p < 0.001); **b**, the AUC of PG II (0.434 ± 0.018, 95% CI: 0.400-0.469) was significantly lower than that of PG I/II (p < 0.001); **c**, the AUC of *H. pylori* antibody (0.574 ± 0.018, 95% CI:0.538- 0.610) was significantly lower than that of PG I/II (p < 0.001)

The AUC of gastric cancer development was higher in PG I/II than in the other biomarkers (Table 1, Fig. 2). The AUC of gastric cancer development was 0.649 ± 0.017 (95% CI: 0.615-0.683) for PG I/II and 0.574 ± 0.018 (95% CI: 0.538-0.610) for *H. pylori* antibody. The optimal cut-off values obtained were 2.5 and 3.0 for PG I/II. When the cut-off value was 2.5 for PG I/II, the optimal values were 71.2% for sensitivity and 52.5% for specificity. When the cut-off value was changed to 3.0 for PG I/II, the optimal values were 86.9% for sensitivity and 39.8% for specificity.

As PG I/II has the highest AUC among the single tests, the AUCs of PG I/II were compared with those of the other methods (Table 2). The AUC of PG I/II with PG I was always higher than that of PG I/II with PG II or *H. pylori* antibody. When the cut-off value was defined as 3.0 for PG I/II, the optimal cut-off point was 65.0 ng/mL for PG I and the optimal values were 81.9% for sensitivity and 42.1% for specificity. The AUC of the combination of PG I/II and PG I was nearly equal to that of PG I/II (*p* = 0.2205).

**Table 2:** Comparison of ROC areas among the combination of 2 biomarkers

PG I/II		PG I	PG II	HP
2.5	ROC area	0.627 ± 0.017	0.619 ± 0.017	0.605 ± 0.018
	(95% CI)	(0.594–0.661)	(0.585–0.652)	(0.571–0.639)
	Optimal cut-off point	65/60	50	5
	Optimal sensitivity	70.2/69.8	71.2	70.8
	Optimal specificity	53.7/54.1	52.5	54.3
3.0	ROC area	0.638 ± 0.017	0.618 ± 0.017	0.594 ± 0.018
	(95% CI)	(0.604–0.672)	(0.604–0.672)	(0.558–0.629)
	Optimal cut-off point	65	50	5.0/6.0
	Optimal sensitivity	81.9	83.9	82.9/82.7
	Optimal specificity	42.1	39.8	42.5/42.7
3.5	ROC area	0.631 ± 0.018	0.618 ± 0.017	0.584 ± 0.082
	(95% CI)	(0.597–0.665)	(0.604–0.672)	(0.548–0.620)
	Optimal cut-off point	65	50	5.0/6.0
	Optimal sensitivity	88.9	91.0	89.9/89.5
	Optimal specificity	31.6	29.2	33.8/34.2

*HP Helicobacter pylori*, *PG* pepsinogen, *ROC* receiver operating characteristic, *CI* confidence interval

**Fig. 3:**
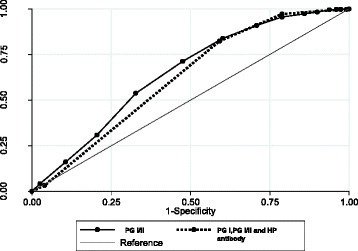
Comparison of AUCs for gastric cancer development among PG I/II, and combined tests using serum PG status and *H. pylori* antibody. Compared with PG I/II, the AUCs of gastric cancer using the combined method with the standard cut-off values (PG I/II = 3.0, PG I = 70.0 ng/mL, and *H. pylori* antibody = 10.0 U/mL) were nearly equal

The predictive sensitivity and specificity of the combination method using PG I/II, PG I, and *H. pylori* antibody were estimated with different titers of *H. pylori* antibody from 1.0 to 10.0 U/mL (Table 3). The cut-off values were defined as 3.0 for PG I/II, 70 ng/mL for PG I, and 10.0 U/mL for *H. pylori* antibody. A “normal” PG level (negative atrophy) and negative for *H. pylori* antibody (negative *H. pylori* infection*)* were defined as negative results. At the standard cut-off value, the sensitivity was 97.2% and the specificity was 21.1%. When the titer was changed from 10.0 to 3.0 U/mL for *H. pylori* antibody, the sensitivity increased slightly but the specificity decreased to half. Thus, with a decrease in the titer of *H. pylori* antibody, the AUCs slightly changed. Compared with PG I/II, the AUCs of gastric cancer using the combined method with the standard cut-off values (PG I/II = 3.0, PG I = 70.0 ng/mL, and *H. pylori* antibody = 10.0 U/mL) were nearly equal (Fig. 3). When the PG I/II value was changed from 3.0 to 2.5 and the PG I value was changed from 70.0 to 65.0 ng/mL in the combination method, the AUCs were similar. The AUCs of gastric cancer development were 0.649 ± 0.017 (95% CI: 0.615-0.683) for PG I/II and 0.622 ± 0.015 (95% CI: 0.593-0.651) for the combination method.

**Table 3:** Comparison of ROC areas among the combination of 3 biomarkers

HP antibody values		PG I/II = 3.0		PG I/II = 2.5	
		PGI = 70	PGI = 65	PGI = 70	PGI = 65
1	ROC area	0.619 ± 0.014	0.623 ± 0.014	0.623 ± 0.015	0.625 ± 0.015
	(95% CI)	(0.591–0.646)	(0.595–0.650)	(0.594–0.653)	(0.596–0.655)
	Sensitivity	99.4	100	99.8	99.8
	Specificity	8.7	4.4	4.6	4.6
3	ROC area	0.620 ± 0.014	0.624 ± 0.014	0.630 ± 0.015	0.632 ± 0.015
	(95% CI)	(0.591–0.648)	(0.596–0.652)	(0.600–0.660)	(0.602–0.662)
	Sensitivity	99.0	99.2	98.6	98.6
	Specificity	11.9	11.9	12.7	12.7
5	ROC area	0.619 ± 0.015	0.624 ± 0.014	0.633 ± 0.016	0.635 ± 0.016
	(95% CI)	(0.591–0.648)	(0.595–0.652)	(0.603–0.665)	(0.605–0.666)
	Sensitivity	98.4	98.4	97.8	97.8
	Specificity	16.9	16.9	17.9	17.7
7	ROC area	0.621 ± 0.015	0.625 ± 0.015	0.634 ± 0.016	0.637 ± 0.016
	(95% CI)	(0.593–0.650)	(0.597–0.654)	(0.604–0.664)	(0.606–0.667)
	Sensitivity	97.4	97.4	96.6	96.6
	Specificity	19.1	19.1	20.1	20.1
10	ROC area	0.622 ± 0.015	0.626 ± 0.015	0.636 ± 0.016	0.638 ± 0.016
	(95% CI)	(0.593–0.651)	(0.597–0.655)	(0.605–0.667)	(0.607–0.669)
	Sensitivity	97.2	97.2	96.4	96.4
	Specificity	21.1	21.1	22.1	22.1

*HP Helicobacter pylori, PG* pepsinogen, *ROC* receiver operating characteristic, *CI* confidence interval

Negative cases were determined when the HP antibody level was below the defined value and the serum PG level was above the defined value of PG I/II or PG I

## Discussion

The association of various risk factors including Cag A, blood type, and lifestyle with gastric cancer development has been investigated, and several risk factors have been shown to have a strong association [6–11]. Although the predication model has been developed based on these results, methods for risk stratification in connection with gastric cancer screening have not been conclusively identified [12, 18]. However, the adaptation of the serum PG and *H. pylori* antibody tests have been anticipated because these methods involve simple blood tests [13–17]. A meta-analysis of prospective cohort studies of gastric cancer development using the combination method of *H. pylori* antibody and serum PG tests with gastric cancer screening has shown that it is possible to stratify the background risks of gastric cancer [21]. In this study, we investigated the best available sensitivity and specificity of the serum PG and *H. pylori* antibody tests for the prediction of gastric cancer development in connection with cancer screening using ROC analysis. However, the AUCs were usually low because the sensitivity was relatively high when the specificity became extremely low. ROC analyses is a graphical technique for assessing the ability of a test to discriminate between those with disease and those without disease [22]. It allows the determination of the cut-off value at which optimal sensitivity and specificity can be obtained and enables the comparison of 2 or more diagnostic tests. Regarding the interpretation of AUC results, a test with an area > 0.9 indicates high accuracy, 0.7–0.9 as moderate accuracy, 0.5–0.7 as low accuracy, and 0.5 as a chance result [22]. In the present ROC analysis, the AUCs for all the methods were below 0.7 even if the highest AUC was obtained when PG I/II was used as a predictive biomarker for gastric cancer development. Based on these definitions, the predictive sensitivity and specificity of gastric cancer development were found to be low in all single tests and combination methods using serum PG and *H. pylori* antibody. Thus, these biomarkers could not discriminate clearly between individuals with and without gastric cancer development in this study.

When the combination method using serum PG and *H. pylori* antibody tests was evaluated in this study, a high sensitivity was obtained; however, the specificity was low. In a previous study using the same dataset for a nested case-control study, a strong association between *H. pylori* infection, gastric atrophy and gastric cancer development was shown. The following odds ratios were obtained when the risk of gastric cancer development was compared with the individuals with both negative *H. pylori* infection and gastric atrophy: 4.2 (95% CI: 2.2-8.0) for the individuals with positive *H. pylori* infection and negative atrophy; 10.1 (95% CI: 5.6-18.2) for the individuals with both positive *H. pylori* infection and gastric atrophy; 4.9 (95% CI: 2.05-12.1) for the individuals with negative *H. pylori* infection and positive gastric atophy [11]. Similar results were obtained from other studies that evaluated the association of *H. pylori* infection, gastric atrophy and gastric cancer development [11, 12, 21]. Although these results confirmed the validity of the strong association for gastric cancer development, and the results supported a high sensitivity for the prediction of gastric cancer development, the possibility of not developing gastric cancer was not assessed and the specificity was ignored. When a prediction model is adopted in clinical practice, it is necessary to provide accurate and discriminating predictions in both situations: with and without gastric cancer development [23]. Therefore, specificity is an important indicator particularity in connection with cancer screening because the target subjects are asymptomatic people. As low specificity translate into an increase in the number of unnecessary examinations, this results in the psychological burden of mislabeling results [20]. When the specificities were calculated on the basis of previous studies which evaluated the association between *H. pylori* infection*,* gastric atrophy, and gastric cancer development, similar results related to sensitivity and specificity were obtained. Based on previous studies related to gastric cancer screening [13–15], the predicative sensitivity and specificity of the combination method using PG I/II, PG I, and *H. pylori* antibody with a standard cut-off value were 94.0% and 34.3%, respectively. Even if other risk factors of gastric cancer were included in the model using PG I/II, PG I, and *H. pylori* antibody, the sensitivity and specificity of gastric cancer development were 96.5% and 28.8%, respectively [18]. Although the basic condition and follow-up times were different in these studies, the predicative accuracy of gastric cancer development was consistently low using the serum PG and *H. pylori* antibody tests. These results have not been given attention because of the lack of a wide perspective in evaluating the balance of benefits and harms in connection with gastric cancer screening. Thus, only sensitivity was similarly evaluated in these studies.

Prognosis was estimated from the risk of future outcomes in individuals based on their clinical and non-clinical characteristics. Prediction performance could be targeted to a high-risk group for cancer screenings and the use of promotion to encourage participation in the screenings. In the case of low-dose CT screening for lung cancer, risk prediction models have been developed based on different variables including smoking and other risk factors [24–27]. The AUCs of these models were 0.67 to 0.88 and these models discriminated the risk of lung cancer adequately. Although *H. pylo*ri infection is a primary cause of gastric cancer development, the serum PG and *H. pylori* antibody tests are insufficient in predicting whether or not an individual has gastric cancer. The aim of an etiological study is to identify particular risk factors attributed to the outcomes. On the other hand, a prediction study provides possible outcomes based on multiple variables associated with the outcome regardless of the cause [28]. In the prediction model, every causal factor is a predictor, but not every predictor is a necessary cause. Because of the possible confusion between an etiological study and a prediction model, biomarkers have been expected to be adopted as cancer screening methods [29, 30]. An accurate prognostic model does not provide any benefits and change the behaviors of the target population of cancer screening if it is not generalizable even though it is verified [31]. In addition, inappropriate use of these biomarkers can lead to a misunderstanding and mismatched labeling of individual risks of cancer. In this study, the highest AUC was obtained in PG I/II, which was also correlated with *H. pylori* infection. Although the ability of PG I/II to discriminate gastric cancer development is limited, there is another possibility of assessing the appropriate screening interval. In HPV screening for cervical cancer, the screening interval can be expanded after a negative result of HPV testing [32, 33]. The diagnosis of atrophy has improved by conventional endoscopy, thus it has been adopted in clinical practice and endoscopic screening. Nomura et al. have reported that endoscopic findings correlated well with PG I/II based on a multicenter prospective study [34]. Hence, endoscopic diagnosis should also be investigated for the prediction of gastric cancer development in connection with gastric cancer screening. Despite the limitation of PG I/II for predicting gastric cancer development, further study on how to effectively utilize it for gastric cancer screening is advantageous.

This study has several limitations. *Firstly,* the background of this study has changed compared with that of other studies in the 1990s. The participants then were recruited in the early 1990s for a large-scale cohort study in Japan. Over the last 2 decades, the incidence of gastric cancer and the infection rate of *H. pylori* have decreased, particularly in younger age groups [35, 36]. Therefore, the present results might not be completely applicable to the current situation. *Secondly*, the study subjects might not be a representative sample of the whole Japanese population. Our study subjects were taken from the dataset of a previous nested case-control study. The subjects were chosen from 97,644 eligible subjects who participated in the survey and blood donation. In the previous study, the participants in the health check-up survey had different socioeconomic statuses and favorable lifestyle profiles, such as smoking less, participating in more physical exercises, and eating more green vegetables and fruits [37]. *Third*, we used a case-control dataset for this analysis. Diagnostic accuracy can be overestimated if the test is evaluated in a group of patients already known to have the disease and in normal patients [38, 39]. The results might also be overestimated. *Fourth*, there was no detailed information regarding the medicine the subjects took for gastric disease. As the health insurance did not cover *H. pylori* eradication during the study period, asymptomatic people had few opportunities to avail of the program. Moreover, a proton pump inhibitor might be also affected by misclassification. *Finally*, we could not completely exclude individuals with gastric cancer at the baseline because the baseline survey included general health check-up, but not endoscopic examination. Therefore, the predictive sensitivity of gastric cancer development might be overestimated.

## Conclusions

In conclusion, the predictive accuracy of gastric cancer development was low with the serum PG and *H. pylori* antibody tests even if these tests were combined. To effectively adopt these biomarkers for gastric cancer screening, high specificity and sensitivity are required. When these tests are included for gastric cancer screening, they should be carefully interpreted in terms of their limitations. Further study is needed on how to adopt risk assessment when using these biomarkers and endoscopic diagnosis in connection with gastric cancer screening.

## Abbreviations

AUC: Area under the curve
*H.pylori*: *Helicobacter pylori*
JPHC study: Japan Public Health Center (JPHC)-based prospective study
PG: Serum pepsinogen
ROC analyses: Receiver operating characteristic analysis
